# Systems Immunology Analysis Reveals an Immunomodulatory Effect of Snail-p53 Binding on Neutrophil- and T Cell-Mediated Immunity in KRAS Mutant Non-Small Cell Lung Cancer

**DOI:** 10.3389/fimmu.2020.569671

**Published:** 2020-12-14

**Authors:** Sarah Musa Hammoudeh, Thenmozhi Venkatachalam, Abdul Wahid Ansari, Riyad Bendardaf, Qutayba Hamid, Mohamed Rahmani, Rifat Hamoudi

**Affiliations:** ^1^ Clinical Sciences Department, College of Medicine, University of Sharjah, Sharjah, United Arab Emirates; ^2^ Sharjah Institute for Medical Research, University of Sharjah, Sharjah, United Arab Emirates; ^3^ Oncology Unit, University Hospital Sharjah, Sharjah, United Arab Emirates; ^4^ Meakins-Christie Laboratories, McGill University, Montreal, QC, Canada; ^5^ Division of Surgery and Interventional Science, University College London, London, United Kingdom

**Keywords:** immunomodulation, snail-p53 binding, non-small cell lung cancer, T-cell mediated anticancer immunity, neutrophil-mediated anticancer immunity, systems immunology, tumor sensitization

## Abstract

Immunomodulation and chronic inflammation are important mechanisms utilized by cancer cells to evade the immune defense and promote tumor progression. Therefore, various efforts were focused on the development of approaches to reprogram the immune response to increase the immune detection of cancer cells and enhance patient response to various types of therapy. A number of regulatory proteins were investigated and proposed as potential targets for immunomodulatory therapeutic approaches including p53 and Snail. In this study, we investigated the immunomodulatory effect of disrupting Snail-p53 binding induced by the oncogenic KRAS to suppress p53 signaling. We analyzed the transcriptomic profile mediated by Snail-p53 binding inhibitor GN25 in non-small cell lung cancer cells (A549) using Next generation whole RNA-sequencing. Notably, we observed a significant enrichment in transcripts involved in immune response pathways especially those contributing to neutrophil (IL8) and T-cell mediated immunity (BCL6, and CD81). Moreover, transcripts associated with NF-κB signaling were also enriched which may play an important role in the immunomodulatory effect of Snail-p53 binding. Further analysis revealed that the immune expression signature of GN25 overlaps with the signature of other therapeutic compounds known to exhibit immunomodulatory effects validating the immunomodulatory potential of targeting Snail-p53 binding. The effects of GN25 on the immune response pathways suggest that targeting Snail-p53 binding might be a potentially effective therapeutic strategy.

## Introduction

The complexity of the tumor micro-environment is attributed to various factors including the different types of cells residing within the tumor micro-environment (i.e. tumor, stromal, and tumor-associated immune cells) (1). These different cell types interact reciprocally, gradually modulating the micro-environment and promoting tumor progression. For instance, the tumor microenvironment modulates the immune response by selectively attracting and repolarizing immune cells (e.g. macrophages and neutrophils) from an anti-tumorigenic to a pro-tumorigenic phenotype (2, 3).

As non-small cell lung cancer (NSCLC) is one of the most common causes of cancer-related death world-wide, targeted and immune therapy strategies are being exhaustively explored for the treatment of this disease (4). Immune-checkpoint inhibitors (ICIs) showed promising results in a group of lung cancer patients (5); however, their efficiency largely depends on the priming and activation state of the immune cells (6). For example, only 14–20% of NSCLC patients were found to benefit from anti-PD-1/PD-L1 therapy (7), as a consequence of the lack of proper immune response dynamics and T-cells priming. Therefore, it is critical to develop immunomodulatory strategies to regulate the activation state of the immune effectors and enhance the efficiency of ICIs (7).

Multiple proteins and signaling pathways were proposed to induce immunomodulatory reciprocal signaling networks between tumor, stromal and immune cells, including p53 and Snail. Inactivation of the tumor suppressor p53 was shown to contribute to tumor progression by augmenting immunotolerance, reducing the infiltration of cytotoxic T-cells and increasing the infiltration of FoxP3^+^ regulatory T cells (8). In addition, in the absence of functional p53, tumor cells promote chronic inflammation and inflammatory cytokines production (e.g. G-CSF, IL6, and CXCL1) through regulation of NF-κB signaling (8, 9). The exacerbation of the chronic inflammation mediated by p53 inactivation promotes the activation of myeloid-derived suppressor cells (e.g. precursors of dendritic cells, macrophages and granulocytes that inhibit T cell response), shifting the nature of the tumor microenvironment further towards a pro-tumorigenic phenotype (10).

Snail on the other hand was found to modulate the secretion of chemokines, such as CXCL2, resulting in the increased infiltration of neutrophils into the tumor micro-environment (11). Moreover, through interacting with CREB-binding protein (CBP), Snail can transcriptionally upregulate the production of various cytokines including TNF-α, CCL2, and CCL5; hence, recruiting tumor-associated macrophages (12). Additionally, Snail was found to induce regulatory T cells differentiation and impair the infiltration of anti-tumor effector cells, through TSP1 and TGF-β production resulting in the resistance to immunotherapy (13).

In 2009, Lee et al. discovered a novel p53 inhibition mechanism instigated by the oncogenic KRAS through inducing Snail-p53 binding and p53 clearance through exocytosis or degradation (14). The compound GN25 was shown to effectively target this binding and, consequently, restore p53 levels and activity in the KRAS mutant cancer cells (15). Therefore, we aimed at investigating the effect of disrupting Snail-p53 binding using GN25 on the modulation of the immune response using systems immunology. To achieve this aim, we analyzed the whole transcriptome of GN25 treated non-small cell lung cancer cell line (A549) using next-generation RNA-seq followed by functional clustering and pathway analysis.

## Materials and Methods

### Cell Culture and Treatment

A549 cells were cultured in RPMI-1640 media supplemented with 10% FBS and 1% penicillin–streptomycin (Sigma) and maintained at 37°C and 5% CO2. The cells were treated with 20 µM GN25 or DMSO vehicle control (NTC) for 24 h and harvested for RNA extraction using RNeasy Mini Kit (Qiagen).

### Quantitative Real-Time PCR Analysis of Gene Expression

Gene-specific cDNA was synthesized using the High-Capacity cDNA Reverse Transcription Kit for RT PCR (Applied Biosystems). qRT-PCR was performed in triplicates with the Maxima SYBR Green/ROX qPCR Master Mix (Thermoscientific) using QuantStudio3 Real-Time PCR instrument (Applied biosystems). qRT-PCR were performed using primers for 18SrRNA, IL8, DUSP1, and CXCL2 as per the sequences in **Table 1**. Gene expression results are presented as mean ± standard error of triplicates.

**Table 1:** Details of primers used for qRT-PCR validation of gene expression.

Gene Symbol	Forward Primer sequence	Reverse Primer sequence	Amplicon Size (bp)	Template gene accession number
18S	TGACTCAACACGGGAAACC	TCGCTCCACCAACTAAGAAC	114	NR_003286
IL8	GAGAGTGATTGAGAGTGGACCAC	CACAACCCTCTGCACCCAGTTT	112	NM_001354840
DUSP1	TCCTGCCCTTTCTGTACCTG	GGACAATTGGCTGAGACGTT	103	NM_004417.4
CXCL2	GGCAGAAAGCTTGTCTCAACCC	CTCCTTCAGGAACAGCCACCAA	127	NM_002089.4

### Whole Transcriptome Analysis

1 ng of RNA extracted from vehicle-treated and GN25-treated A549 cells was analyzed using targeted whole RNA-seq with AmpliSeq whole transcriptome on S5 system (Thermo Fisher Scientific). The SuperScript VILO cDNA synthesis kit (Invitrogen) was used to synthesize barcoded CDNA libraries; which were further amplified using Ion AmpliSeq transcriptome human gene expression kit (Thermo Fisher Scientific). Taqman library quantitation kit (Applied Biosystems) was used to evaluate the quality of the libraries. Pooled libraries were then amplified using emulsion PCR on Ion One Touch2 instruments (OT2) and enriched using Ion One Touch ES as per manufacturer’s instructions. RNA-sequencing of the libraries was done using Ion S5 XL Semiconductor sequencer on Ion 540 Chip (Life Technologies).

### Bioinformatics Analysis

RNA-seq data was analyzed using the Ion Torrent Software Suite version 5.4. Alignment was carried out using the Torrent Mapping Alignment Program (TMAP). TMAP is optimized for aligning the raw sequencing reads against reference sequence derived from hg19 (GRCh37) assembly. To maintain specificity and sensitivity, TMAP was used to implement a two-stage mapping approach. First, four alignment algorithms, BWA-short (BWA, http://bio-bwa.sourceforge.net) (16), BWA-long (17), SSAHA (18), and super-maximal exact matching (19) were employed to identify a list of candidate mapping locations. A further alignment process is performed using the Smith-Waterman algorithm (20) to find the final best mapping. Raw read counts of the targeted genes were performed using samtools (samtools view –c –F 4 –L bed_file bam_file). The quality control including the number of expressed transcripts is checked after Fragments per Kilobase Million (FPKM) normalization. Differentially expressed gene analysis was carried out using a modification of the NOISeq algorithm (21) with raw read counts from RNASeq data. The cut-off chosen for NOISeq is q = 0.8 based on the noise content of the samples.

### *In Silico* Functional Analysis

Over and under expressed genes were subject to functional analysis using unsupervised hierarchical clustering based on Gene Ontology analysis. We analyzed the functional clustering of the differentially expressed genes using Metascape (22) and Ingenuity pathway analysis (IPA) platforms (23) annotation tools. To identify the specific effect of GN25 on the different types of immune cells, we cross matched the differential transcriptome with gene ontology sets retrieved using AmiGO 2 database (**Table S1**). Heatmap and bar plot representations were generated using R (version 3.6.0). We queried our differential immune expression signature for overlap with other immunomodulatory compounds through the L1000 Characteristic Direction Signature Search Engine (https://amp.pharm.mssm.edu/L1000CDS2) developed by the Mount Sinai Center for Bioinformatics (24).

### Western Blot

Cells were lysed with RIPA lysis buffer containing 50 mM Tris Base, 150 mM NaCl, 1% sodium deoxycholate, 0.1% SDS, 1% Triton X-100, and supplemented with 1X protease inhibitor cocktail (Sigma). Protein concetration was then quantified using Pierce™ BCA Protein Assay Kit (ThermoFisher). 20µg of each sample sample were separated on 12.5% SDS polyacrylamide gel and transblotted onto polyvinylidene difluoride (PVDF) membrane (Biorad). Membranes were blocked with 5% Bovine Serum Albumin prepared in 1X TBST then incubated with the primary antibodies overnight at 4°C. Anti-p53 rabbit polyclonal antibody (A0263, abclonal) and anti-β-actin mouse monoclonal antobody (A5441, Sigma) were applied at a dilution of 1:1,000. The secondary antibodies, Anti-mouse IgG, HRP-linked Antibody #7076 (Cell Signaling, 1:3,000) and Anti-rabbit IgG, HRP-linked Antibody #7074 (Cell signling, 1:3,000), were applied to the membranes for 1 hour at room temperature. Chemiluminescence was detected using Pierce™ ECL Western Blotting Substrate (ThermoFisher Scientific) and developed using the ChemiDoc™ imaging system (Biorad). Quantification of bands was analyzed by Image LabTM software (Biorad).

### PBMC Isolation, Co-Culture, and Flow Cytometry

Peripheral blood mononuclear cells (PBMCs) were isolated from fresh blood samples collected from four healthy donors following the approval of the ethical committee at University Hospital Sharjah. The research ethics approval code for this study is UHS-HERC-033-02042020. The PBMCs were isolated using histopaque gradient separation (Sigma). PBMCs were labeled with 4 µM carboxyfluorescein succinimidyl ester (CFSE, Invitrogen, USA) for 8 min at room temperature and washed with ice cold RPMI 1640 medium (completed with 10% FBS and 1% penicillin-streptomycin). PBMCs were then directly co-cultured with a monolayer of A549 cells treated with 0, 5, and 10 µM of GN25 in complete RPMI 1640 medium at a seeding ratio of 1:1 (5×10^4^ cells per well in a 12-well cell culture plate). PBMCs were harvested on the third day of culture and stained with anti-Human CD3-AlexaFluor 700 (clone OKT3, eBiosciences, Invitrogen, USA). Stained PBMCs were then acquired using BD FACS Aria III flow cytometer (BD Biosciences, USA) and BD FACS Diva software.

### Statistical Analysis

Two-tailed t-test was conducted to statistically analyze the significance of the gene expression data and T-cell activation data; the significance was taken to be p <0.05. All statistical analyses were performed using GraphPad Prism (version 5.01).

## Results

### Validation of GN25 Effect on Cell Cycle and Cytoskeleton Reorganization

**Figure 1:**
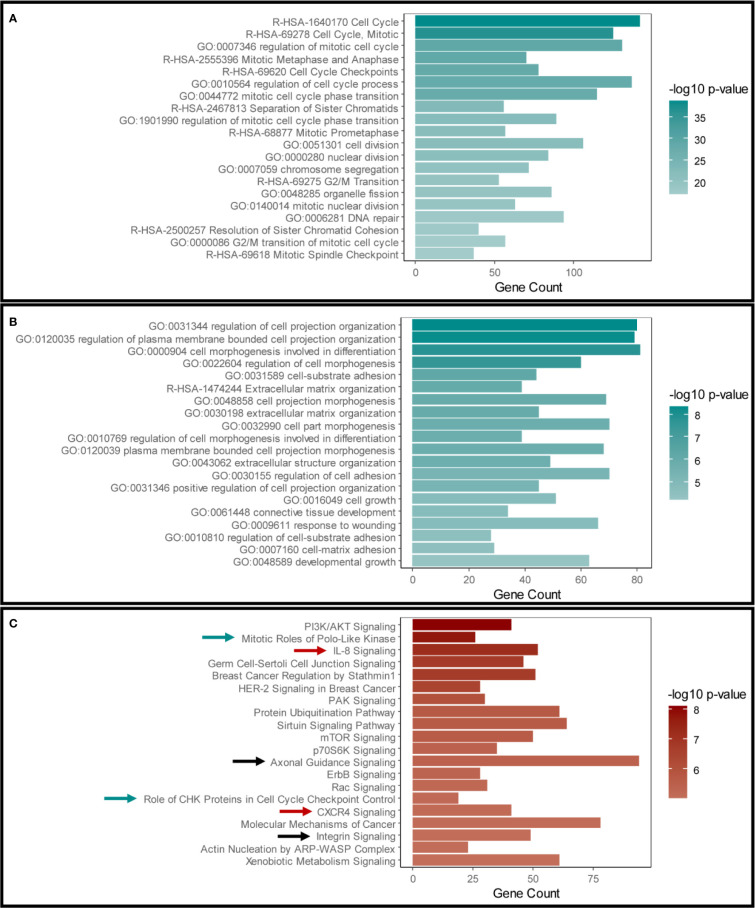
Validation of Snail inhibition and p53 restoration in GN25 treated cells. The top 20 enriched pathways and functional clusters in **(A)** the upregulated genes and **(B)** downregulated genes in the GN25 treated cells analyzed using Metascape annotation tool **(C)**. Top 20 pathways enriched in the differentially expressed genes in GN25 treated cell (both up- and down-regulated genes) using IPA. Green arrows indicate cell cycle related pathways; black arrows indicated cell morphogenesis related pathways; and black arrows indicate immune response related pathways.

Our initial aim was to examine the effects of disrupting Snail-p53 binding using GN25 on the gene expression profile of the NSCLC cell line A549. The transcriptome of GN25 treated NSCLC cells was enriched for transcripts implicated in cell cycle regulation, phase transition, cell division, and DNA damage and repair (**Figure 1A**). Furthermore, many of these transcripts belong to p53 signaling cascade, involved in the regulation of cell cycle and DNA repair (**Table S2**). The upregulation and enrichment of these transcripts confirms the restoration of p53 activity in response to disrupting its binding to Snail by GN25.

On the other hand, GN25 treatment resulted in the downregulation of transcripts involved in cell projection organization, cell morphogenesis and differentiation, extracellular matrix organization, and regulation of cell adhesions (**Figure 1B**). The downregulation of these transcripts is a potential consequence of Snail inhibition exerted by GN25, in concordance with previous findings (25).

We cross-validated these enrichment results analyzed using Metascape with those analyzed using Ingenuity Pathway Analysis (IPA) (**Figure 1C**). Analysis results from both tools confirmed the significant effect of GN25 treatment on cell cycle and cell projections organization, subsequently to Snail inhibition and p53 restoration. Intriguingly, multiple immune pathways were enriched in both analyses, suggesting a potential effect of the disruption of Snail-p53 binding on the immune response (**Figure 1C**).

We next carried out *in vitro* validation to confirm the restoration of p53 in response to GN25 treatment at the protein level. In concordance with the findings of Lee at al. (15), we observed a significant upregulation (F.C.) of p53 levels in A549 cells treated with 20 µM GN25 (**Figure S1A**). Moreover, we observed a reduction in the proliferation of A549 cells in response to GN25 treatment (**Figures S1B, C**).

### Enrichment in Immune Response Genes and Pathways in GN25 Treated NSCLC Cells

**Figure 2:**
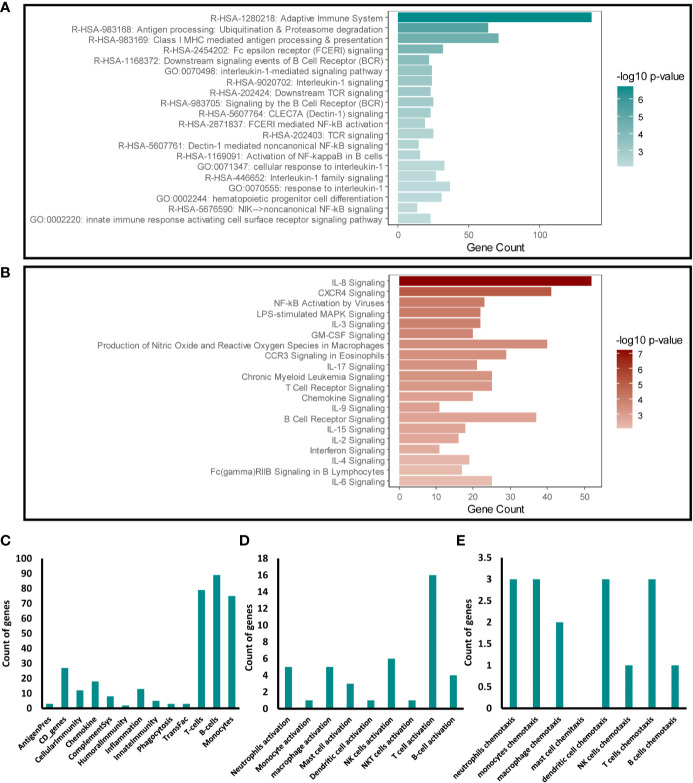
Immunomodulatory effect of Snail-p53 binding inhibition. Enriched representation of the top 20 immune response pathways and functional clusters identified in analysis conducted using **(A)** Metascape and **(B)** IPA. The count of intersecting genes from the GN25 treated cells with the **(C)** Immunome set **(D)**, immune cells activation gene ontology sets, and **(E)** immune cells chemotaxis gene ontology sets.

We next focused our analysis on the enriched immune response transcripts and pathways to further examine the effects of disrupting Snail-p53 binding on the immunomodulatory potential of NSCLC cell line, A549. Targeted Enrichment of our pathway analysis results from Metascape and IPA for immune response pathways, uncovered a significant effect of GN25 treatment on signaling pathways mediated by cytokines (e.g. IL-8, IL-1, IL-17), immune receptors (e.g. TCR, BCR, and FcϵR), and NF-κB (**Figures 2A, B**).

To further investigate the specific effect of GN25 on the immune response, we examined the overlap between the differential transcriptome of GN25 treated NSCLC cells and gene ontology sets linking to the regulation and activation of the different arms of the immune system. Our analysis showed a substantial overlap with T-cell, B-cell and Monocyte gene sets from the Immunome gene ontology set (26–28) (**Figure 2C**). Enrichment of T cell regulatory transcripts was further confirmed through the substantial overlap with the T cell activation and chemotaxis gene sets retrieved from AmiGO 2 database (**Figures 2D, E**). Overlap, although less substantial, was observed as well with the activation and chemotaxis gene sets of other immune cells including neutrophils, monocytes, and NK cells. These results suggest a potential effect of Snail-p53 binding disruption on the immunomodulatory capacity of NSCLC cells on immune response mediated by different immune effectors, including T cells.

### GN25 May Increase the Immunomodulatory Effect of NSCLC Cells on Myeloid Cells, Neutrophils, T-Cells Mediated Immune Response

**Figure 3:**
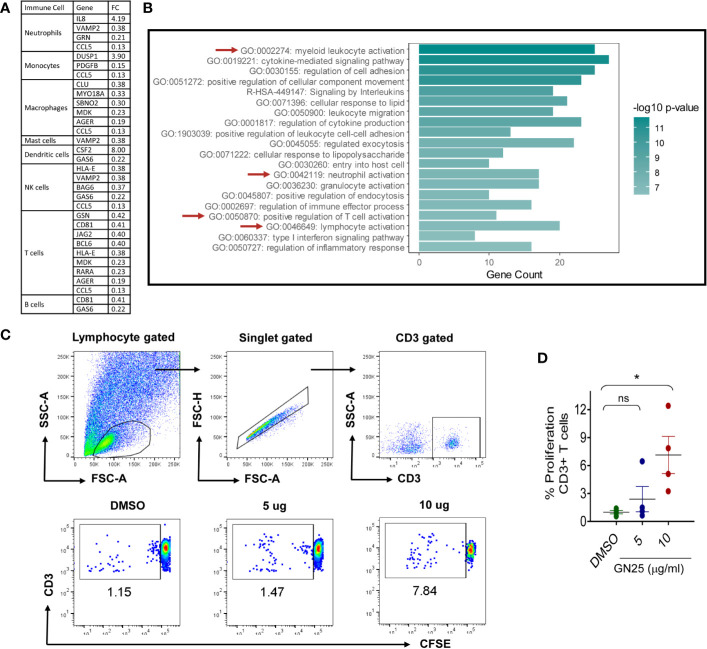
Most significantly enriched immune pathways within the immunomodulatory signature of GN25 **(A)**. The differentially expressed genes (Fold changes cut-off >2 and <0.5) enriched in the gene ontology data sets for the activation and chemotaxis of each immune cell type **(B)**. Pathway analysis of Differentially expressed genes enriched in the Immunome and activation/chemotaxis Gene ontology sets of each immune cell type. Pathway analysis done using Metascape **(C)**. Representative FACS plots showing the percent of CFSE+ CD3+ cells co-culture with A549 cells treated with 5 and 10 µM GN25 as well as vehicle control **(D)**. dotplot representation of mean percentage ± SEM of CD3+ cells proliferation in response to co-culture with A549 cells treated with 5 and 10 µM GN25 as well as vehicle control. Data shown are from four healthy individuals. * represents p-value <0.05.

Despite the marked effect of GN25 treatment on the enrichment of T cell regulatory transcripts, the effect of GN25 encompassed transcripts implicated in the regulation of other elements of the immune response (e.g. neutrophils, Monocytes, and NK cells). To further assess the significance of the effect of GN25 on the remaining elements of the immune response, we filtered the differentially expressed genes (fold change >2 or <0.5) that overlapped with the immune response gene ontology sets for functional clustering analysis, including IL8, CD81, BCL6, and DUSP1 (**Figure 3A**; example of the filtered genes).

Although the results of the functional clustering analysis showed a general enrichment of leukocytes activation, migration and regulation pathways, a significant enrichment was observed for myeloid leukocytes (GO:0002274), neutrophil (GO:0042119) and T cell (GO:0050870 and GO:0046649) regulatory pathways in comparison to the other immune cell types (**Figure 3B**). The constitutive enrichment of T cell regulatory pathways at the different levels of analysis as well as the enrichment of the neutrophil and myeloid leukocyte regulatory IL-8 signaling pathway suggest a substantial effect of Snail-p53 binding disruption of myeloid leukocytes, neutrophils and T cells mediated immunity.

Intriguingly, the suppression of some of the identified immune response genes, including CD81, might contribute to the suppression of cancer cells migration and invasive capacity (29). Moreover, treatment with GN25 is suppressing BCL6 which was shown to alternatively promote cancer cells survival through exerting a suppressive effect on DNA damage sensing proteins including p53 (30).

### Direct Immunomodulatory Effect of GN25 Treated A549 Cells on CD3+ Lymphocytes

We next carried out *in vitro* validation of the suggested immunomodulatory effect of GN25 treated NSCLC cells on T and B cells. We directly co-cultured freshly isolated PBMCs with GN25 treated A549 cells and monitored CD3+ PBMCs for proliferation using CFSE as a marker of cell activation. We observed a significant upregulation of cell proliferation in response to the co-culture with A549 cells treated with 10 µM GN25 (**Figures 3C, D**). These findings support the enhancement of the immunomodulatory capacity of NSCLC cells in response to the disruption of Snail-p53 binding as suggested by the in silico analysis of the RNA-seq data.

### Snail-p53 Signaling Modulates the Immune Response Potentially Through NF-κB Signaling and Related Regulatory Pathways

**Figure 4:**
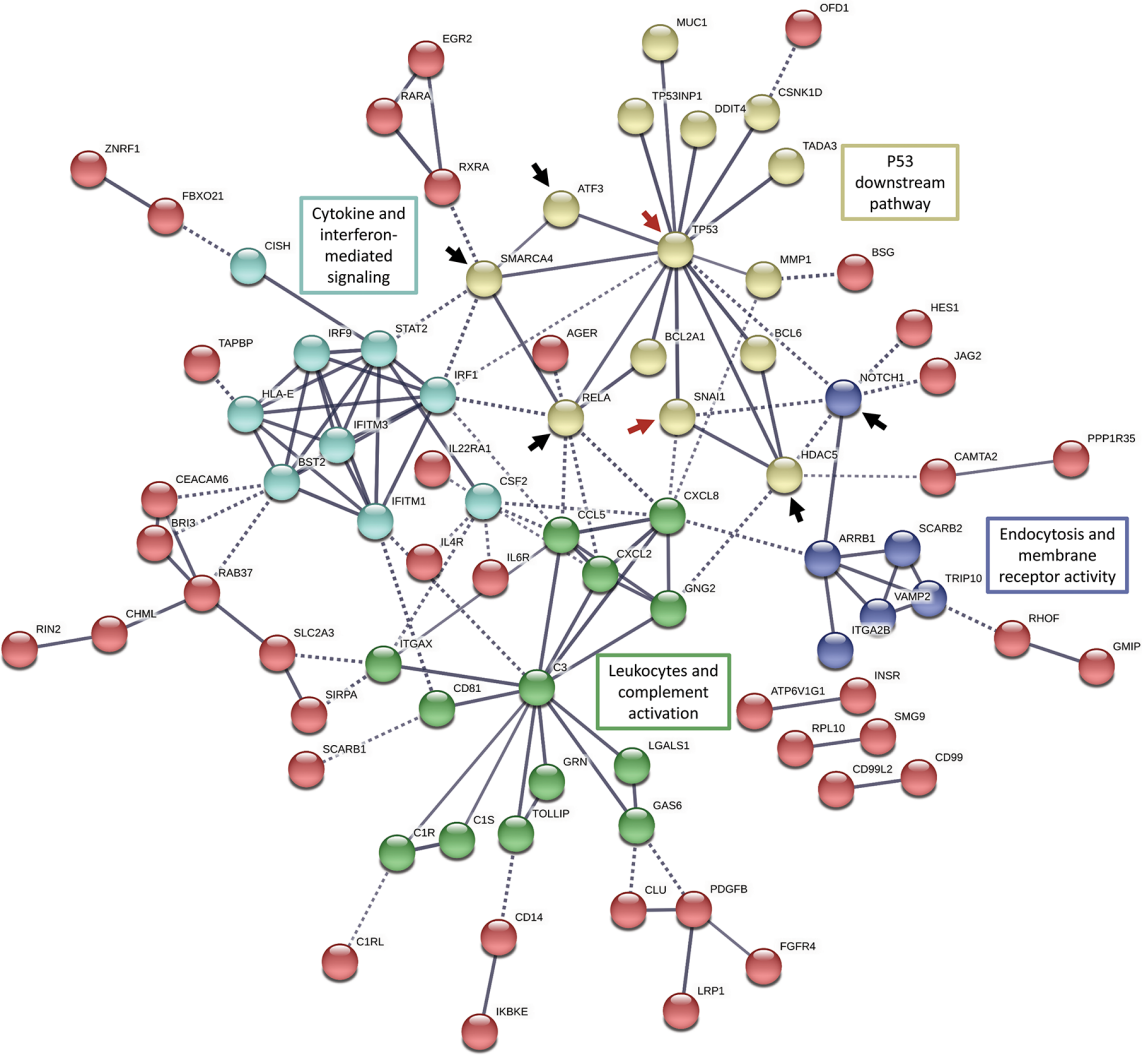
String functional protein association networks analysis in all the differentially expressed genes (FC >2 or <0.5) enriched in the immune gene ontology sets. Red arrows indicate elements of our target molecules, p53 and Snail; black arrows indicate potential regulatory nodes induced by Snail-p53 binding to modulate immune response pathways.

The functional clustering and pathway analysis of the differentially expressed immune-related genes revealed an enrichment for transcripts contributing to major regulatory signaling pathways such as ERK1/2, AP1, Notch, JAK-STAT, NF-κB, and MAPK signaling pathways. Henceforth, the observed immunomodulatory effects of Snail-p53 binding disruption could be an end result of targeting these regulatory signaling pathways. Therefore, we aimed at further analyzing our differentially expressed immune genes for potential regulatory networks using String functional protein association networks analysis (**Figure 4**).

In concordance with the enrichment of NF-κB signaling pathway (hsa04064), we observed that the NF-κB pathway active subunit RELA, occupies a central node in the functional network connecting p53 to cytokines-mediated signaling and leukocytes activation. The transcription factor ATF3 appears to be induced by p53 signaling as well to regulate cytokine-mediated signaling and leukocytes activation.

Moreover, the network analysis suggests that histone deacetylase 5 (HDAC5) propagates p53 and Snail signaling to regulate leukocytes and complement activation, confirming thereby the enrichment of the histone modification pathways in our previous analysis (e.g. GO:0031056: regulation of histone modification). Snail-p53 binding may potentially regulate endocytosis, transport across the plasma membrane, and membrane receptors signaling through NOTCH1, an element of the NOTCH signaling pathway enriched in our previous analysis (GO:0007219: NOTCH signaling pathway).

Altogether, these findings suggest that Snail-p53 binding disruption displays an immunomodulatory role pertaining to its upstream effect on regulatory signaling pathways such as NF-κB signaling.

### Immunomodulatory Genes Are Enriched in the Top 20 Up- and Down-Regulated Genes in GN25 Treated A549 Cells

**Figure 5:**
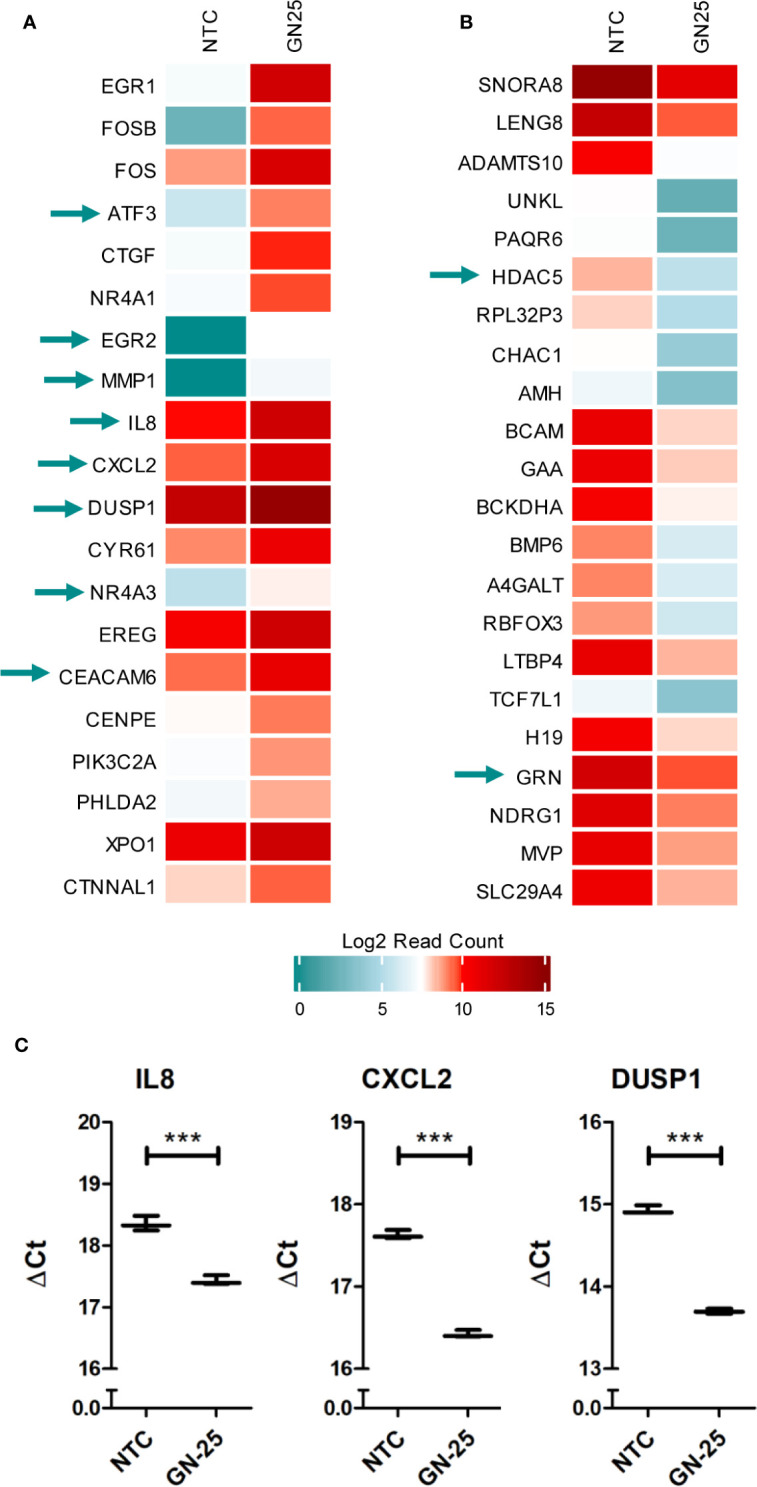
Immune genes enrichment in the top differentially expressed genes. heatmap representation of the log2 Read counts of the **(A)** top 20 upregulated genes and the **(B)** top 20 downregulated genes in GN25 treated cells in comparison to the vehicle negative control. Green arrows indicate genes that intersect with the differentially expressed genes enriched in the immune gene ontology gene sets **(C)**. Gene expression validation using qRT-PCR of immune response genes identified in the RNA-seq analysis including IL8, CXCL2, and DUSP1. *** represents p-value <0.005.

To further confirm the substantiality of the immunomodulatory potential of Snail-p53 binding disruption, we examined the top 20 upregulated and downregulated genes, for overlap with the differential immune expression profile of GN25 treated NSCLC cells. We found that 25% of the top 20 upregulated and downregulated genes overlapped with the queried immune signature (**Figures 5A, B**).

GN25 treatment resulted in the significant upregulation of important regulatory hubs of adaptive and innate immunity, such as ATF3 and DUSP1, which play critical roles in processing upstream signals from various stimuli, to regulate downstream inflammatory and immune response pathways (31–33). Moreover, GN25 appears to upregulate the expression of the nuclear receptor subfamily 4 group A member 1 (NR4A1), an anti-inflammatory protein that regulates NF-kB signaling and inflammatory cytokines production (e.g. IL-12) (34). These results further confirm the considerable effect of GN25 on the modulation of immune-related pathways and processes.

Despite the inhibitory effect of GN25 on various immune response elements, we observed the significant upregulation of some pro-tumorigenic immune modulators potentially as a compensatory feedback mechanism. For instance we observed the upregulation of the carcinoembryonic antigen-related cell adhesion molecule 6 (CEACAM6), an emerging target for anti-cancer therapies due to its important pro-tumorigenic role in lung cancer (35). Moreover, CXCL2, a chemokine that contributes to cancer metastasis as well as pro-tumorigenic immunomodulation (36), is upregulated in our GN25 treated samples. The upregulation of these genes suggests that the lung cancer cells are employing backup mechanisms to offset the inhibitory effects Snail-p53 inhibition on the immune response and cell movement.

The significant upregulation of some of these genes (IL8, DUSP1, and CXCL2) in GN25 treated A549 NSCLC cells was further validated *in vitro* using qRT-PCR (**Figure 5C**).

### Partial Correlation of GN25 Immune Expression Profile to Those of Compounds With Immunomodulatory Effects

We next aimed at assessing the potential of employing the disruption of Snail-p53 binding as an immunomodulatory therapeutic strategy. We queried the immune expression profile of GN25 treated NSCLC cells against the expression signatures in the L1000 Characteristic Direction Signature Search Engine L1000CDS2 (24) to identify compounds with concordant immunomodulatory effects (**Table 2**).

**Table 2:** Compounds with signatures overlapping with the immunomodulatory signature of GN25.

Compound/Drug	Overlap score	Cell-line	Dose	Time
PD-0325901	0.1223	A375	0.04 um	24 h
selumetinib	0.1223	MCF10A	10 um	24 h
WZ-4-145	0.1223	MCF10A	10 um	24 h
radicicol	0.1223	MCF10A	1.11 um	24 h
AS-605240	0.1151	A375	3.33 um	24 h
afatinib	0.1151	MCF10A	3.33 um	24 h
gefitinib	0.1151	MCF10A	10 um	24 h
TG101348	0.1079	A549	11.1 um	6.0 h
PLX-4720	0.1007	A375	0.37 um	24 h
GSK-2126458	0.1007	MCF10A	1.11 um	24 h

The top two query candidates were the MEK1/2 inhibitors, PD-0325901 and selumetinib, reported to modulate interferon signaling and chemokines production to reduce inflammation (37). Another candidate found to overlap with the queried immune signature is the heat shock protein 90 (Hsp90) inhibitor radicicol; established to reduce inflammation by suppressing cytokines and IFN-gamma production and macrophages stimulation (38, 39). The PI3Kγ inhibitor AS-605240, another top candidate, was found to suppress lung carcinoma inflammation and the secretion of pro-inflammatory cytokines (e.g. IL-17, IFN-γ, IL-22, GM-CSF, IL-6, IL-4, and IL-13) (40–43).

The immunomodulatory signature of GN25 was found to overlap with multiple additional compounds which similarly possess immunomodulatory effects such as gefitinib, erlotinib (44), JAK2 inhibitor TG101348 (45), and mTOR inhibitor GSK-2126458 (46). The concordance in the immune expression patterns of GN25 and these compounds, supports the potential utility of targeting Snail-p53 binding as a potential immunomodulatory therapeutic strategy.

## Discussion

The tumor microenvironment gradually undergoes modulations to promote tumor growth through the reciprocal signaling between the different cell types (i.e. tumor, stromal, and immune cells) encapsulated within the tumor vicinity. Oncogenic reprogramming of signaling pathways expands the tumor cells’ capacity to modulate the immune response and induce pro-tumorigenic chronic inflammation (47). Therefore, reprogramming the immune response is being heavily investigated as a therapeutic strategy. A number of regulatory proteins are currently being studied for their immunomodulatory effects, including p53 and Snail. In this paper, we aimed at exploring the immunomodulatory potential of disrupting the Snail-p53 binding induced by oncogenic KRAS in NSCLC cells.

Our analysis of the differential transcriptome induced by the disruption of Snail-p53 binding confirmed the enrichment of transcripts contributing to various immune response pathways, including signaling pathways mediated by cytokines, immune receptors, and NF-κB. Moreover, the disruption of Snail-p53 binding exerted a substantial effect of the regulation, activation, and chemotaxis of multiple immune effectors. The effect of targeting Snail-p53 binding was most significantly observed on the expression of transcripts contributing to neutrophils- and T cell- mediated immunity, such as IL-8, BCL6, and CD81. Previous studies revealed that p53 and Snail, each independently, plays an important role in regulating tumor infiltration of T-cell and neutrophil, respectively (8, 11). Our findings that GN25 modulated neutrophils and T-cells tumor responses may result from the combination of p53 activation and Snail inhibition and suggest that GN25 might be as effective as combined treatment with p53 activators and Snail inhibitors in NSCLC.

Moreover, analysis of the differential transcripts implicated in immune response pathways showed an enrichment for multiple major regulatory pathways including ERK1/2, AP1, Notch, JAK-STAT, NF-κB, and MAPK signaling pathways. Elements of these pathways (e.g. RELA and NOTCH1) act as central nodes that propagate changes in Snail and p53 activity to the different arms of the immune response. Henceforth, we speculate that the disruption of Snail-p53 binding exhibits immunomodulatory effects in NSCLC cells as an end result of modulating upstream regulatory signaling pathways. These findings are concordant with the previously observed p53-mediated regulation of cytokines production and inflammation through the modulation of NF-κB signaling (8, 9). However, further functional studies are required to identify the precise signaling cascades targeted by the disruption of Snail-p53 binding to modulate the different components of the immune response.

We further validated the potential of targeting Snail-p53 binding as a potential immunomodulatory therapeutic strategy by confirming the concordance in the immune expression patterns of GN25 and well-documented immunomodulatory compounds (e.g. PD-0325901, selumetinib and radicicol). Some of these compounds such as selumetinib and radicicol, were found to complement and sensitize tumors to immune checkpoint inhibitors such as anti-CTLA4 and anti-PD1 therapies (48–50). Therefore, we speculate that targeting Snail-p53 binding using GN25 would similarly potentiate the antitumor activities of ICIs.

Cancer cells exhibit high capacity in maintaining their oncogenic state by utilizing an extensive network of feedback loops and compensatory mechanisms, resulting in the development of drug resistance (51, 52). We observed evidence of these compensatory mechanisms in our studies. For instance, we observed the upregulation of pro-metastatic genes (e.g. CEACAM6) to compensate for the GN25 treatment. Moreover, GN25 treatment upregulated CXCL2 expression as a potential mechanism to recover the pro-tumorigenic immune microenvironment by recruiting tumor promoting myeloid cells (36). These findings provide an elaborate example on the intricate plasticity and adaptability of cancer cells to resist external influences resulting in the inefficiency of various therapeutic approaches, including immunomodulatory treatments.

However, since our investigations are mostly based on systems immunology analysis of transcriptomic data, the outcomes described in this paper are mostly speculations on the immunomodulatory role of Snail-p53 binding. Further mechanistic and functional validation *in vitro* and *in vivo* is required to substantiate our claims on the immunomodulatory potential of disrupting Snail-p53 binding; to comprehensively understand the dynamics and mechanism of action of this potential therapeutic strategy; and to investigate the potential mechanisms of compensatory resistance that may develop against this approach. Moreover, whilst other studies showed the p53 restorative effect of GN25 on p53 wild type cells, future work is needed to assess the replicability of our findings in other NSCLC cells lines as well as primary cells. The differentially expressed immune biomarkers in our data (e.g. IL-8, BCL6, and CD81) can be further investigated as potential biomarkers for the prediction of patients’ response to the proposed chemotherapeutic approach and the potential acquisition of drug resistance.

In conclusion, we used a systems immunology approach to investigate the immunomodulatory potential of the disruption of Snail-p53 binding using GN25. Our analysis of the whole transcriptome data confirmed the enrichment of transcripts implicated in immune response signaling pathways, especially the concomitant regulation of neutrophils- and T cell- mediated immunity, as indicated by the differential expression of IL8, BCL6, and CD81. The data presented in the study suggests that the disruption of Snail-p53 binding could be further investigated as an immunomodulatory therapeutic strategy to sensitize immune effectors for enhanced patient response to immune therapies (e.g. ICIs such as anti-CTLA-4 and anti-PD-1/PD-L1).

## Data Availability Statement

The datasets presented in this study are provided in the **Supplementary Material** (**Table S3**) for GN25- and vehicle- treated samples.

## Ethics Statement

The studies involving human participants were reviewed and approved by University Hospital Sharjah (Ethical approval number UHS-HERC-033-02042020). The patients/participants provided their written informed consent to participate in this study.

## Author Contributions

SH and RH were responsible for the conception, design, and development of the methodology. SH, TV, and AA were responsible for the practical application of the methodology and data acquisition. SH and RH were responsible for the bioinformatics analysis and data interpretation. SH, MR, RB, QH, and RH were responsible for the writing and reviewing the manuscript. RH, MR, QH, and RB were responsible for the supervision of the study. All authors contributed to the article and approved the submitted version.

## Funding

This project is funded by Al-Jalila Foundation (Grant No: AJF201741 and AJF2018090), Boehringer Ingelheim (Grant No: 120102) and Sheikh Hamdan Award for Medical Science Research (Grant No: MRG/108/2018). The funder bodies were not involved in the study design, collection, analysis, interpretation of data, the writing of this article or the decision to submit it for publication.

## Conflict of Interest

The authors declare that the research was conducted in the absence of any commercial or financial relationships that could be construed as a potential conflict of interest.
