# Supplementary material for: Systems Immunology Analysis Reveals an Immunomodulatory Effect of Snail-p53 Binding on Neutrophil- and T Cell-Mediated Immunity in KRAS Mutant Non-Small Cell Lung Cancer
Source: Front Immunol. 2020 Dec 14;11:569671. doi: 10.3389/fimmu.2020.569671 (PMC7768232; doi:10.3389/fimmu.2020.569671)
Supplement: Supplementary file 1 [file Table_1.docx]

Supplementary Material

# Supplementary Tables

**Table S1**. Accession of Gene ontology sets retrieved from AmiGO 2 for the analysis of immune cell types specific gene enrichment

| **Accession** | **Term** |
| --- | --- |
| **GO:0042119** | Neutrophil activation |
| **GO:0030593** | Neutrophil chemotaxis |
| **GO:0042117** | Monocyte activation |
| **GO:0002548** | Monocyte chemotaxis |
| **GO:0042116** | Macrophage activation |
| **GO:0048246** | Macrophage chemotaxis |
| **GO:0045576** | Mast cell activation |
| **GO:0002551** | Mast cell chemotaxis |
| **GO:0001773** | Myeloid dendritic cell activation |
| **GO:0002407** | Dendritic cell chemotaxis |
| **GO:0030101** | NK cell activation |
| **GO:0035747** | NK cell chemotaxis |
| **GO:0051132** | NK T cell activation |
| **GO:0042110** | T cell activation |
| **GO:0010818** | T cell Chemotaxis |
| **GO:0042113** | B cell activation |
| **GO:0035754** | B cell chemotaxis |

**Table S2.** Enriched pathways relating to p53 regulatory signalling in GN25 treated A549 cells identified through Metascape functional clustering and pathway analysis

| **Enriched Pathway** | **Log p-value** | **Log (q-value)** | **Count Ration** |
| --- | --- | --- | --- |
| **R-HSA-3700989** Transcriptional Regulation by TP53 | -7.82 | -5.72 | 52/365 |
| **GO:0072331** signal transduction by p53 class mediator | -6.71 | -4.75 | 40/268 |
| **R-HSA-5633007** Regulation of TP53 Activity | -4.78 | -3.08 | 25/160 |
| **GO:1901796** regulation of signal transduction by p53 class mediator | -4.78 | -3.08 | 27/180 |
| **R-HSA-6791312** TP53 Regulates Transcription of Cell Cycle Genes | -4.60 | -2.91 | 12/48 |
| **GO:0030330** DNA damage response, signal transduction by p53 class mediator | -4.56 | -2.89 | 19/107 |
| **R-HSA-69541** Stabilization of p53 | -4.47 | -2.81 | 13/57 |
| **R-HSA-69563** p53-Dependent G1 DNA Damage Response | -4.39 | -2.75 | 14/66 |
| **R-HSA-69580** p53-Dependent G1/S DNA damage checkpoint | -4.39 | -2.75 | 14/66 |
| **R-HSA-69610** p53-Independent DNA Damage Response | -4.22 | -2.61 | 12/52 |
| **R-HSA-69613** p53-Independent G1/S DNA damage checkpoint | -4.22 | -2.61 | 12/52 |
| **GO:0006977** DNA damage response, signal transduction by p53 class mediator resulting in cell cycle arrest | -3.89 | -2.34 | 12/56 |
| **R-HSA-6804756** Regulation of TP53 Activity through Phosphorylation | -3.83 | -2.29 | 16/92 |
| **R-HSA-6804115** TP53 regulates transcription of additional cell cycle genes whose exact role in the p53 pathway rema | -2.20 | -1.01 | 5/20 |

# Supplementary Figures


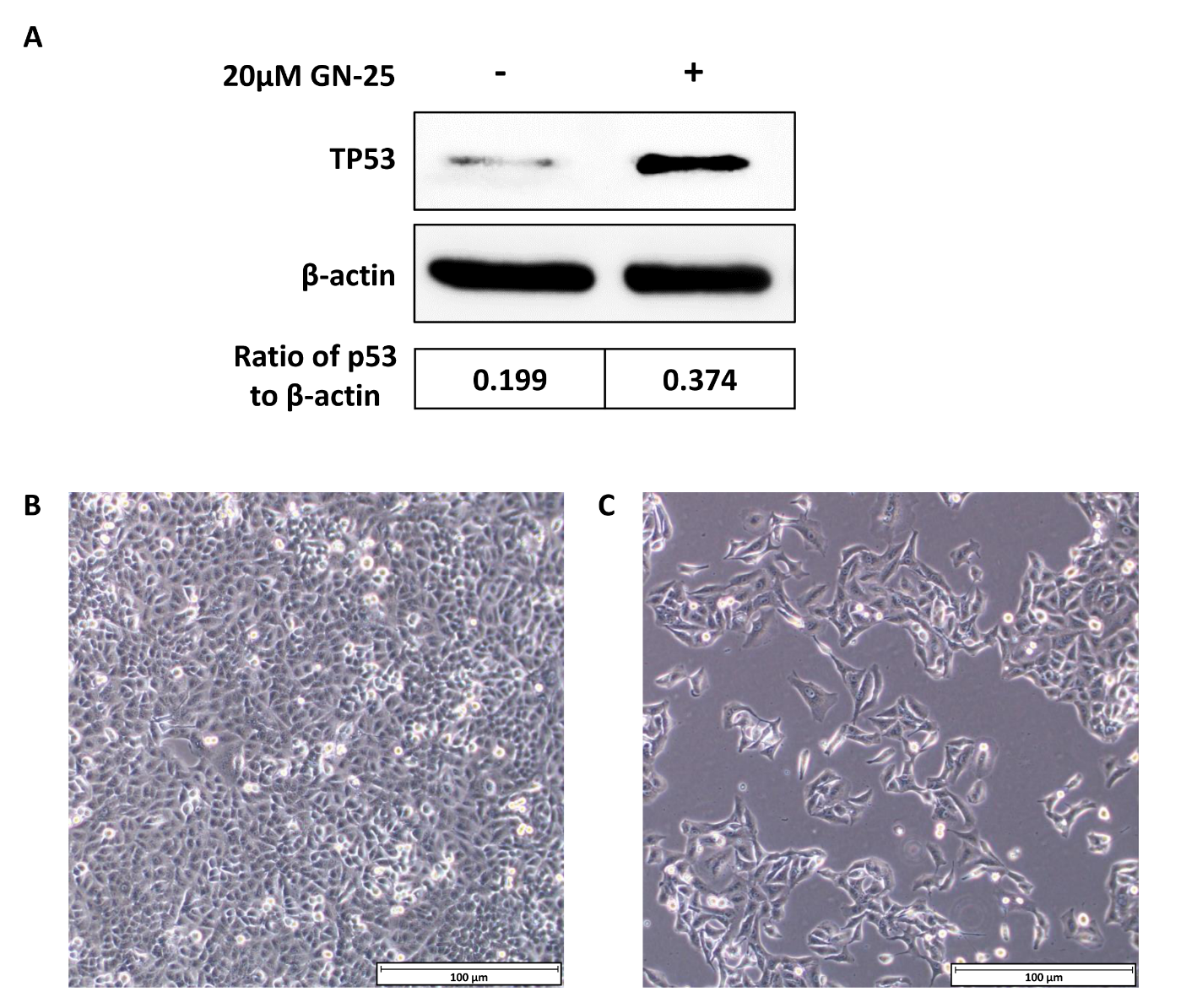


**Figure S1.** **(A)** Western blot analysis of p53 expression in A549 cells treated with vehicle control (DMSO) and 20µM GN25. Phase contrast light microscopy images of A549 cells treated with **(B)** vehicle control and **(C)** 20µM of GN25 for 72 hours. Scale bars representing 100µm. Images acquired with 20X magnification through Olympus IX53 inverted microscope.
